# Modeling of signaling crosstalk-mediated drug resistance and its implications on drug combination

**DOI:** 10.18632/oncotarget.11745

**Published:** 2016-08-31

**Authors:** Xiaoqiang Sun, Jiguang Bao, Zhuhong You, Xing Chen, Jun Cui

**Affiliations:** ^1^ Zhongshan School of Medicine, Sun Yat-Sen University, Guangzhou, 510080, China; ^2^ School of Mathematical and Computational Science, Sun Yat-Sen University, Guangzhou, 510000, China; ^3^ School of Life Science, Sun Yat-Sen University, Guangzhou, 510275, China; ^4^ School of Mathematical Sciences, Beijing Normal University, Beijing, 100875, China; ^5^ School of Computer Science and Technology, China University of Mining and Technology, Xuzhou, 221116, China; ^6^ School of Information and Electrical Engineering, China University of Mining and Technology, Xuzhou, Jiangsu, 221116, China; ^7^ Collaborative Innovation Center of Cancer Medicine, Sun Yat-Sen University, Guangzhou, 510060, China

**Keywords:** signaling crosstalk, drug resistance, drug combination, synergism

## Abstract

The efficacy of pharmacological perturbation to the signaling transduction network depends on the network topology. However, whether and how signaling dynamics mediated by crosstalk contributes to the drug resistance are not fully understood and remain to be systematically explored. In this study, motivated by a realistic signaling network linked by crosstalk between EGF/EGFR/Ras/MEK/ERK pathway and HGF/HGFR/PI3K/AKT pathway, we develop kinetic models for several small networks with typical crosstalk modules to investigate the role of the architecture of crosstalk in inducing drug resistance. Our results demonstrate that crosstalk inhibition diminishes the response of signaling output to the external stimuli. Moreover, we show that signaling crosstalk affects the relative sensitivity of drugs, and some types of crosstalk modules that could yield resistance to the targeted drugs were identified. Furthermore, we quantitatively evaluate the relative efficacy and synergism of drug combinations. For the modules that are resistant to the targeted drug, we identify drug targets that can not only increase the relative drug efficacy but also act synergistically. In addition, we analyze the role of the strength of crosstalk in switching a module between drug-sensitive and drug-resistant. Our study provides mechanistic insights into the signaling crosstalk-mediated mechanisms of drug resistance and provides implications for the design of synergistic drug combinations to reduce drug resistance.

## INTRODUCTION

Drug resistance is often an inevitable obstacle in drug efficacy of the targeted therapeutics for cancer patients [1–3]. Many paradigms of mechanisms have been revealed to explain the drug resistance at molecular, cellular and microenvironmental levels [4–9]. Intracellular signaling pathways link the cell's genome to the extracellular microenvironment [10]. Various types of crosstalk among such signaling pathways enable dynamic modulations of signal transduction network [11]. Whether and how the signaling dynamics mediated by crosstalk contributes to the drug resistance are intriguing and remain to be systematically investigated.

Various cancer studies for targeted therapy suggested that the signaling crosstalk could provide subtle posttranslational activation of signaling pathways which can bypass the stress of the therapeutic target and further modulate the expression patterns of oncogenes [12–14]. For example, a crosstalk (ERK inhibiting HGFR) exists between EGF/EGFR/Ras/Raf/MEK/ERK pathway and HGF/HGFR/PI3K/AKT pathway [15]. Relief of profound crosstalk inhibition of HGFR signaling by drugs (or inhibitors) targeting BRAF attenuates the drug efficacy in BRAFV600E melanomas [15, 16]. Moreover, crosstalk among signaling pathways may occur at multiple levels of signal transduction [17, 18]. For instance, EGFR/HER2 signaling pathways can crosstalk with other receptor tyrosine kinases, including MET, IGF1R, FGFR and EphA2, at the receptor level via the shared downstream targets of EGFR/HER2. At mediator level, the activation of mutated BRAF crosstalks with the upstream receptors such as EGFR and can independently activate downstream effectors [19].

Biological signaling pathways in cells are known to often interact with each other via extensive signaling crosstalk. Crosstalk-linked signaling modules [20] or motifs [21, 22] may carry out key functions of the signaling network. Therefore, it's instructive to analyze the key kinetics of small signaling networks to get more mechanistic understanding on the properties and functions of typical modules. Previous studies have developed some motif-based models to investigate the dependence of drug efficacy on the network topology. Behar, M. et al. [10] examined how the combinations of signaling hub topologies and dynamical signals affect the relative sensitivity of the stimulus response to the pharmacological perturbations. Zhang, Y. et al. [23] computationally analyzed how variations in pairs of parameters synergistically (or antagonistically) affect the response in a series of small molecular network motifs. Van, W.R. et al. [24] investigated non-monotonic input-output relation arising from simple network topologies by analyzing two simple interacting linear signaling pathways that carry two different signals with different physiological responses. However, these works did not investigate the causal relationship between the architectures of crosstalk in the signaling network and drug resistance.

In this study, we investigated the contributions of signaling crosstalk to the drug resistance using module-based kinetic modeling approach. Motivated by experimental evidence of drug resistance mediated by crosstalk between signaling pathways, we developed kinetic models for eight small molecular networks with typical crosstalk modules. We investigated the signaling dynamics of proteins in the modules with or without the drug treatment. Our simulations demonstrated that various types of signaling crosstalk could afford rich dynamics of proteins in the modules, and that crosstalk inhibition could diminish the response of signaling output to the stimuli. Moreover, we found that signaling crosstalk affects the relative sensitivity of drugs, and we further identified some crosstalk modules that could result in resistance to the targeted drugs. In addition to the structure of the crosstalk, the kinetic parameters of crosstalk, i.e., the strength of the crosstalk, might also be important in determining the network dynamics. Therefore, we further analyzed the role of the crosstalk strength in switching a module between drug-sensitive and drug-resistant responses.

As a promising strategy to overcome drug resistance, combination therapy has been suggested to improve the effects of targeted therapeutics [25–29]. As demonstrated by Yin, N., et al. [30], the synergism or antagonism of drug combinations largely depends on the network topology. Moreover, the synergism of drug combinations is not always identical to effectiveness. Therefore, to both synergistically and effectively reduce drug resistance, it's important to select drug targets in the signaling network for rationally designing optimal drug combinations [29, 31, 32]. We therefore quantitatively evaluated both the relative efficacy and synergism of drug combinations. For the modules that were resistant to the targeted drug, we identified pairs of drug targets that could not only increase the relative drug efficacy but also exhibit synergistic effect when they were targeted in a combinatorial manner. Our results indicated that the downstream proteins or transcriptional factors in the crosstalk-linked signaling pathways might be important potential targets of drug combination to reduce the resistance of targeted therapeutics.

## RESULTS

### Experimental evidence and computational modeling

**Figure 1:**
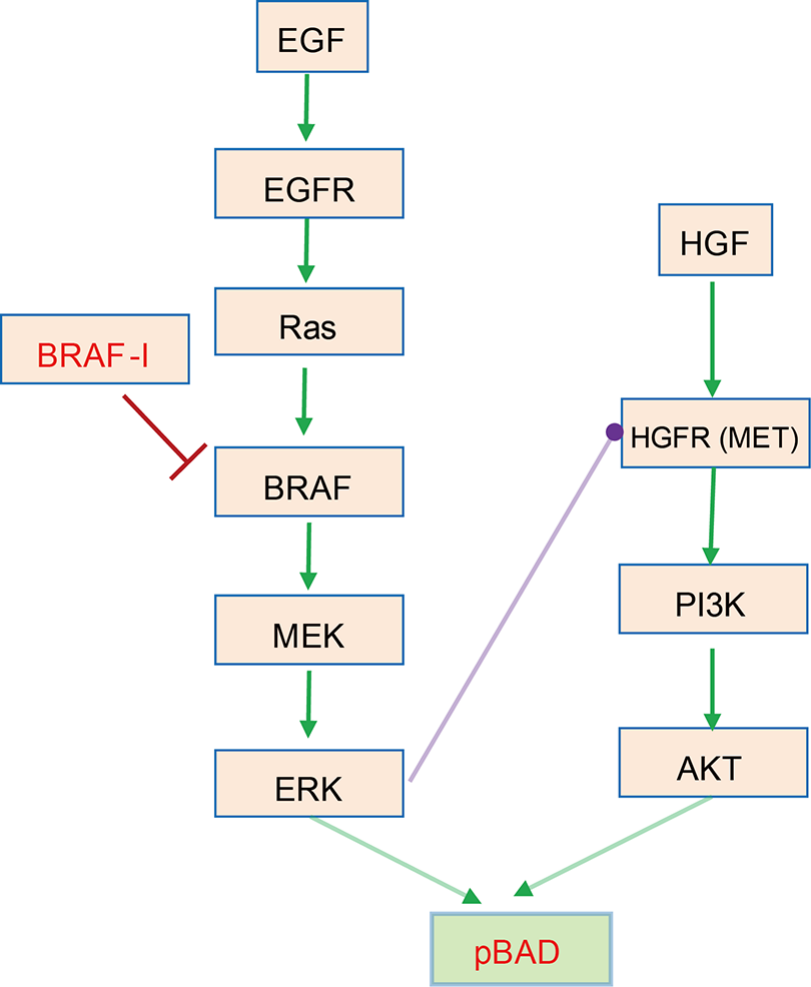
Drug resistance mediated by crosstalk inhibition between EGF/EGFR/Ras/Raf/MEK/ERK and HGF/HGFR/PI3K/AKT pathways in BRAFV600E melanomas [15] These two pathways promote the phosphorylation of BAD [16] that plays a critical role in anti-apoptosis of many types of tumor cells [33]. High levels of ERK-dependent crosstalk inhibition suppress HGFR signaling and PI3K activation. BRAFV600E is sensitive to RAF inhibitor that potently inhibits BRAF and ERK signaling, resulting in relief of ERK-dependent crosstalk inhibition and reactivation of HGFR signal transduction. This, in turn, sustains the activation and function of downstream BAD signaling and attenuates the drug efficacy in BRAFV600E melanomas [15].

We first examined a realistic case of widely studied signaling pathways as shown in Figure 1. Stimulated by growth factors EGF and HGF respectively, EGFR/Ras/Raf/MEK/ERK and HGFR/PI3K/AKT pathways [15] converge to phosphorylate BAD [16] that plays a critical anti-apoptotic role in many types of tumor cells [33]. A crosstalk inhibition from ERK to HGFR exists between these two pathways. Relief of profound crosstalk inhibition of HGFR signaling by drugs (or inhibitors) targeting BRAF attenuates the drug efficacy in BRAFV600E melanomas [19]. As such, this signaling crosstalk contributes to drug resistance.

**Figure 2:**
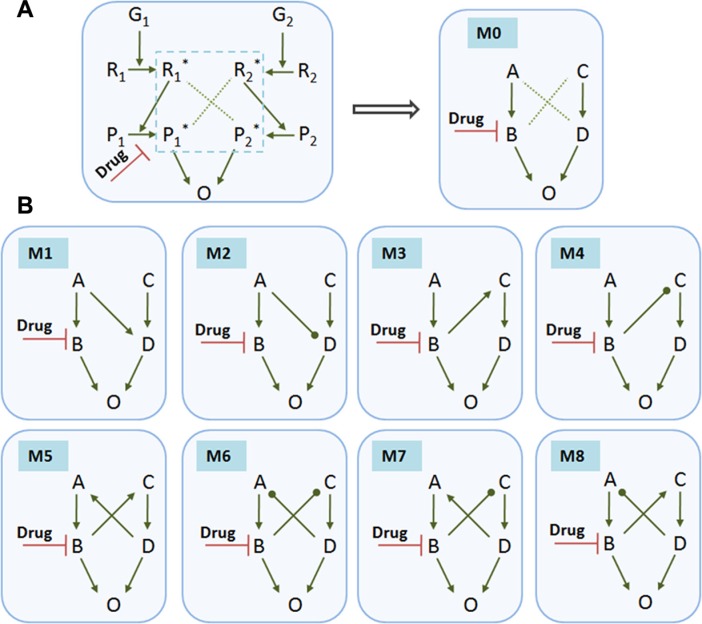
Typical crosstalk-linked signaling modules Various modules contain different types of crosstalk between two signaling pathways. (**A**) A small network of enzyme-catalyzed cascades with crosstalk between two pathways stimulated by two growth factors G_1_ and G_2_, respectively. G_1_ promotes the phosphorylation of protein R_1_ into R_1_^*^, and G_2_ promotes the phosphorylation of protein R_2_ into R_2_^*^. The signals from R_1_^*^ or R_2_^*^ activates P_1_ or P_2_, respectively. The activated P_1_^*^ and P_2_^*^ converge to promote the activation or expression of the signaling output, O. The drug is considered to inhibit the activation of P_1_. This study focuses on the effects of crosstalk between two pathways in the core module (blue dashed line) of the signaling network on the drug efficacy. We reduced this module to a simplified form represented as module 0 (M0) as shown in the right panel. This basic module 0 without crosstalk was chosen as a control case for comparison. (**B**) Various modules that contain different types of signaling crosstalk: M1, A activating D; M2, A inhibiting D; M3, B activating C; M4, B inhibiting C; M5, B activating (**C** and **D**) activating A; M6, B inhibiting C, and D inhibiting A; M7, B inhibiting C, and D activating A; M8, B activating C, and D inhibiting A. For simplicity, we only considered these 8 typical modules containing one- and two-crosstalk links that were commonly found in intracellular signaling networks.

Motivated by this experimental evidence, we then asked a more general question about how signaling dynamics of crosstalk-linked modules affect the drug efficacy and which types of signaling crosstalk could result in the drug resistance. For simplicity, we investigated two interacting pathways stimulated by two growth factors G_1_ and G_2_, respectively, as shown in Figure 2A (left panel). G_1_ promotes the phosphorylation of protein R_1_ into R_1_^*^, and G_2_ promotes the phosphorylation of protein R_2_ into R_2_^*^. The signaling cascades from R_1_ and R_2_activate P_1_ and P_2_, respectively. The activated P_1_^*^ and P_2_^*^ converge to the output, O, and promote its activation or expression. The drug was considered to inhibit the activation of P_1_. Since our study focuses on the effects of crosstalk between two signaling pathways in the core module (blue dashed line) on the drug efficacy, we represented this module of enzyme-catalyzed cascades as an abbreviated reaction form, module 0 (M0). As shown in the right panel of Figure 2A, these two pathways were simply and generally represented as G1-A-B and G2-C-D, where G_1_ and G_2_ are two growth factors, A and C represent upstream receptors or proteins, and B and D represent downstream proteins or transcriptional factors.

We then considered 8 typical modules that contain different types of signaling crosstalk (Figure 2B): M1, A activating D; M2, A inhibiting D; M3, B activating C; M4, B inhibiting C; M5, B activating C, and D activating A; M6, B inhibiting C, and D inhibiting A; M7, B inhibiting C, and D activating A; M8, B activating C, and D inhibiting A. For simplicity, only these 8 typical modules were considered, with the assumption of network symmetry. The basic module 0 without crosstalk was chosen as a control case for comparison. A drug inhibiting B was incorporated for all modules. We used kinetic equations (see Materials and Methods) to simulate the temporal activations of proteins in each signaling module.

### Signaling crosstalk affects stimuli-response dynamics

**Figure 3:**
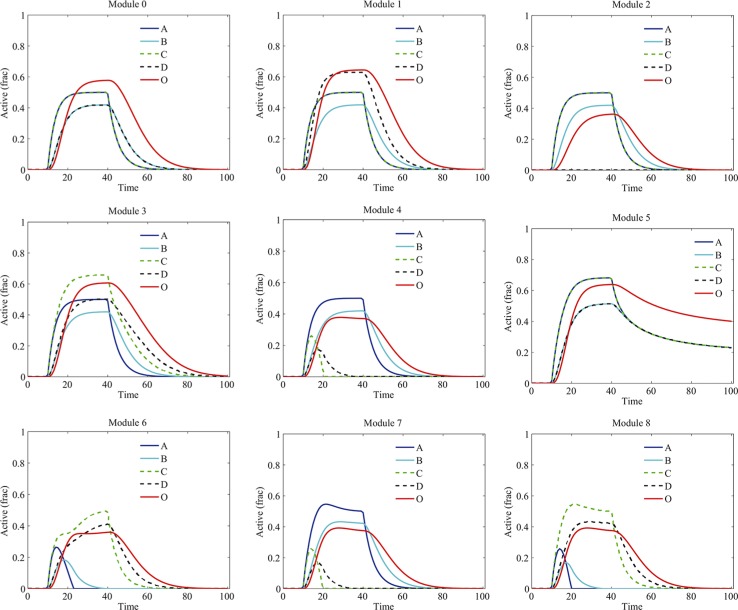
Temporal dynamics of proteins in various modules without drug treatment Different types of crosstalk resulted in different dynamics of proteins and signaling output. “Active (frac)” indicates fraction of activation of each protein.

We investigated the dynamics of components in various signaling modules in response to the stimuli of G1 and G2 without drug treatment (Figure 3). Different types of crosstalk resulted in different dynamics of proteins and signaling output. In the basic module 0, due to the assumption of symmetry, the time courses of A and C were identical, so were that of B and D. Their activation levels were elevated up by the stimuli and then decreased due to their degradation after the elimination of the stimuli. In the module 1, due to the crosstalk activation from A to D, the level of D was higher than that in the module 0. Whereas in the module 2, the activation level of D was diminished due to the crosstalk inhibition from A to D. In the module 3, the activation levels of C and D were higher than that in the module 0, the opposite situation was observed for the module 4 where the activation of C as well as D was inhibited by B. Interestingly, the activations of proteins in module 5 exhibited persistence even after the elimination of the stimuli, which was due to the mutual crosstalk activation between two pathways. In the module 6, we assumed the crosstalk inhibition from D to A was stronger than that from B to C, so the pathway A-B-O was repressed. In the modules 7 and 8, incoherent mutual crosstalk between two pathways enhanced one side of signaling but inhibited another side. Overall, the levels of signaling outputs in the modules with crosstalk inhibition (modules 2, 4, 6, 7 and 8) were lower than that in other modules (modules 0, 1, 3 and 5).

### Signaling crosstalk affects drug sensitivity

**Figure 4:**
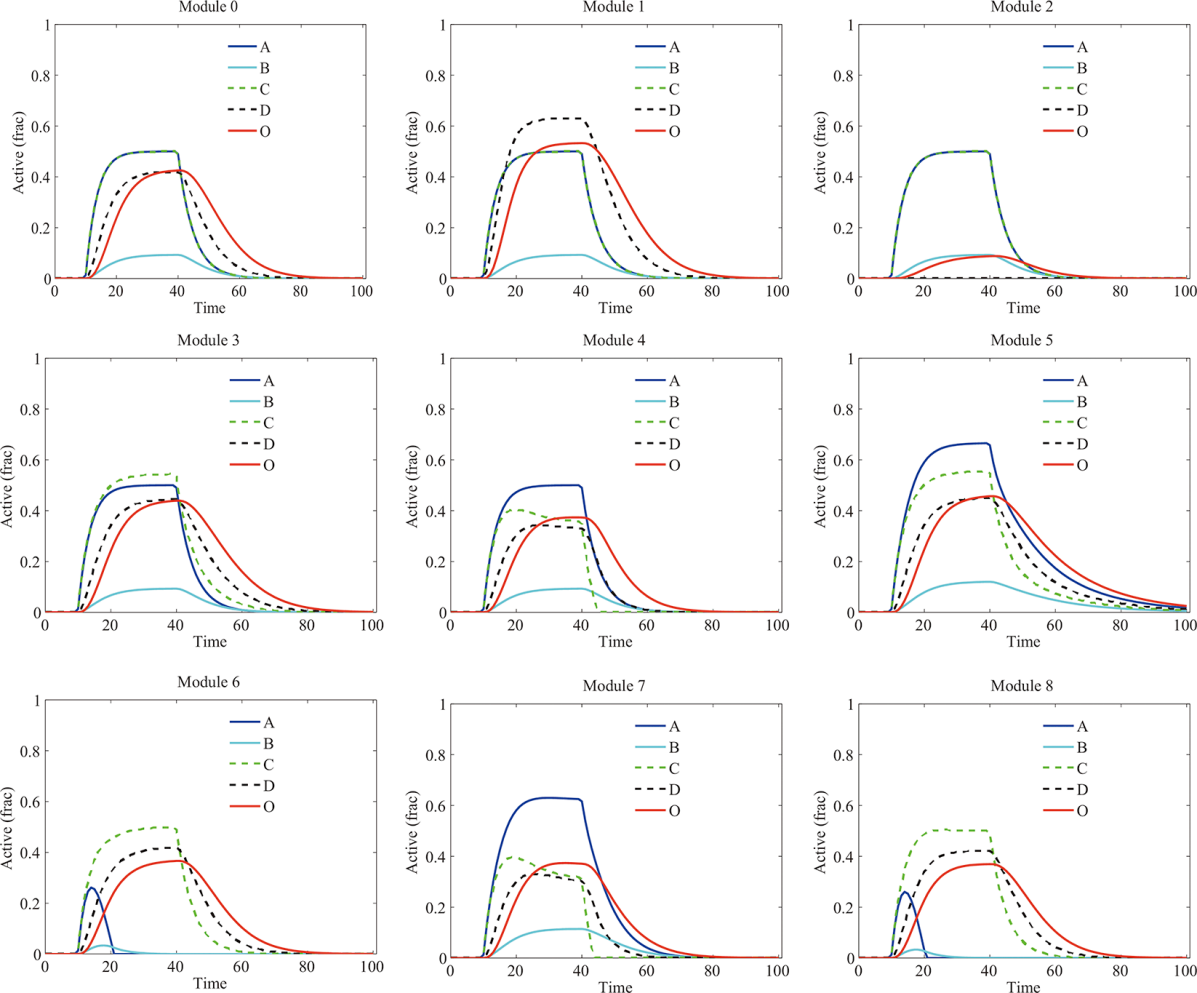
Temporal dynamics of proteins in various modules under the treatment of B-targeting drug Different types of signaling crosstalk have different impacts on the drug-induced dynamics of proteins and signaling output. “Active (frac)” indicates fraction of activation of each protein.

Next, we incorporated the drug effects into the model and investigated the influence of signaling crosstalk on the kinetics of proteins in various modules. In our simulation, component B was chosen as the drug target. Different types of signaling crosstalk have different impacts on the drug-induced dynamics of proteins and signaling output (Figure 4). Under the drug treatment, the activation of B was significantly decreased in all the modules. Other proteins presented various distinct dynamics in different modules. Particularly, in module 2, due to the crosstalk inhibition of D by A, both the activation of B and D were downregulated thus the signaling output was significantly decreased. Notably, the persistence of signaling activation in module 5 without drug treatment (Figure 3) was disappeared due to the drug treatment.

**Figure 5:**
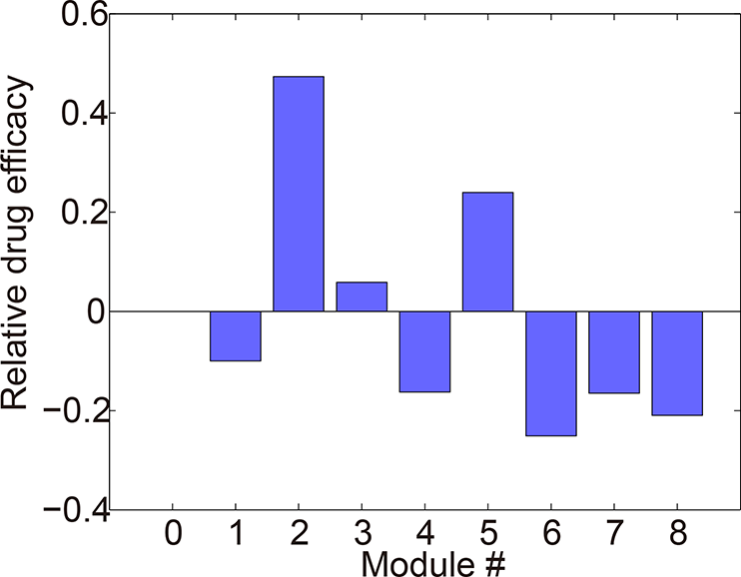
Relative drug efficacies of 8 modules under the treatment of B-targeting drug Modules 1, 4, 6, 7 and 8 exhibited lower drug efficacies as compared to the basic module 0, indicating that the crosstalk in these modules could render cells insensitive to the drug treatment.

We calculated relative drug efficacy (see Materials and Methods) for 8 different modules. The drug efficacies in modules 1, 4, 6, 7 and 8 were less than 0 (Figure 5), indicating that the drug-induced relative reduction of output in these modules were lower than that in the basic module 0. This result demonstrated that the signaling crosstalk in these modules could dampen the efficacy of B-targeting drug and thus contribute to the drug resistance.

We then take a close look at the kinetics of these drug-resistant modules (1, 4, 6, 7 and 8) to get more mechanistic insights into the crosstalk-mediated drug resistance. The module 1 represents a common molecular mechanism underlying the drug resistance of targeted therapy. Although component B in one side of the pathway was inhibited by the drug, the bypassing signaling from A to D increased the signaling in the other pathway thus maintained the signaling output. The module 4 contains a crosstalk inhibition from B to C. The B-targeting drug relieved this inhibition and thus restored the signals of C and D and the activation of the output. The crosstalk-mediated dynamic adaptation of the cell signaling system to the drug treatment provides an important mechanism underlying drug resistance, which is consistent with Figure 1 and validated by experiments in [19]. In addition, our simulation (Figure 5) also suggested that the mutual crosstalk inhibition in module 6 could result in drug resistance, which is consistent with a realistic module as shown in Figure S1 and the experimental results in [34]. Similar mechanism holds for modules 7 and 8 where crosstalk inhibition exists.

We also examined the relative efficacy of B-targeting drug under various forms of input stimuli (Figure S2). In the simulation, the stimuli of G1 and G2 were varied alone or simultaneously to various types. Modules 1, 4, 6, 7 and 8 are more prone to resisting B-targeting drugs (Figure S3), which is consistent with the above results (Figure 5).

### *In silico* screening of drug combinations

The rational application of drug combinations has been suggested to be beneficial for combating drug resistance [27]. Given the limitation of the molecularly targeted agents currently available, the traditional experimental screening approach is expensive, time consuming and practically unfeasible. *In silico* screening of drug combinations provides an alternative rational approach [35, 36]. Here we employed the kinetic model to examine whether combinatorial drugs perform better than the single drug in the context of drug resistance based on various module structures. We used the relative drug efficacy and synergism as two indices to evaluate the drug combinations for various modules.

**Figure 6:**
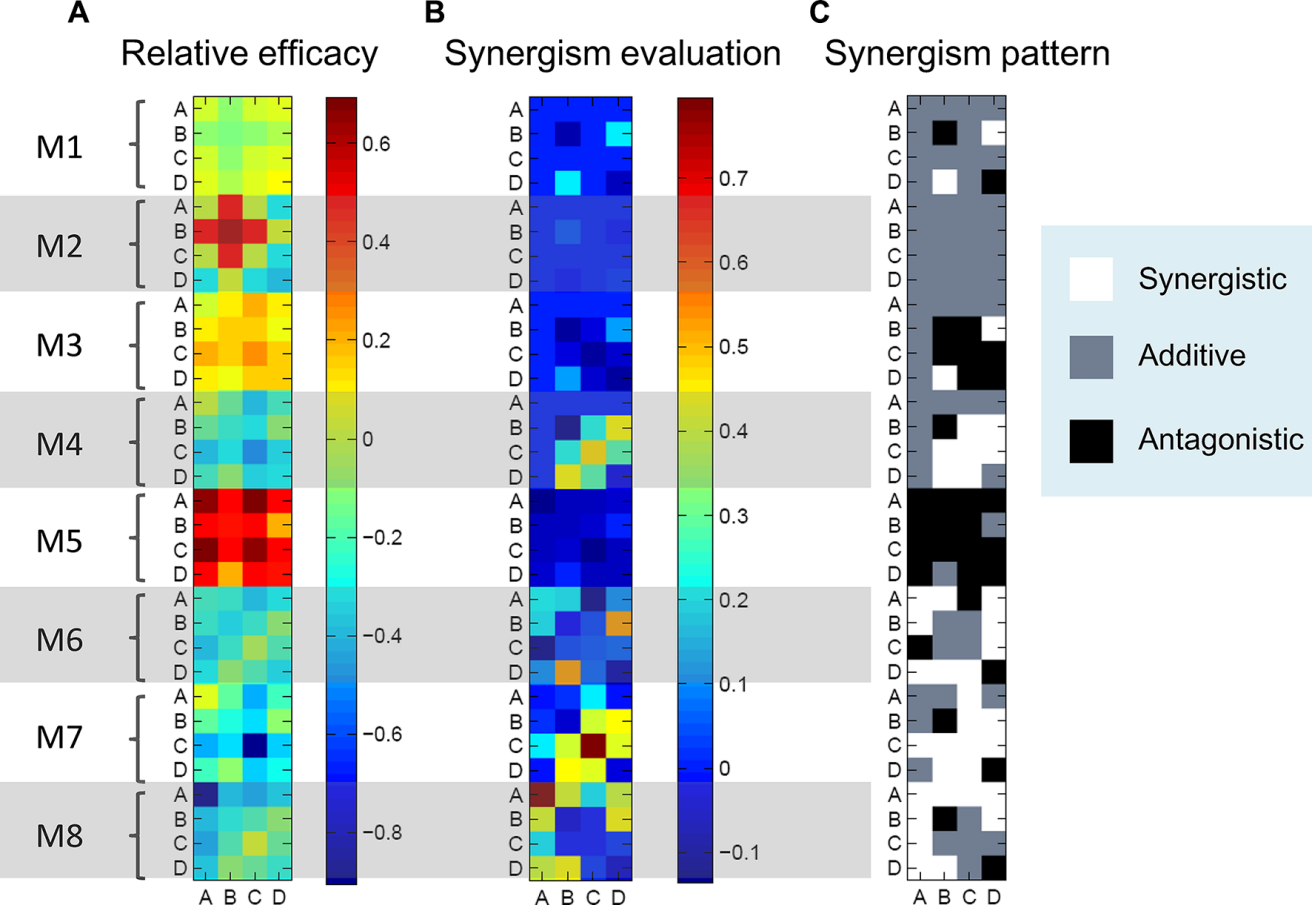
Drug combination evaluation (**A**) Relative efficacies of drug combinations (A, B, C and D) for different modules. The efficacy of drug combination was dependent on the topology of crosstalk-linked signaling modules. (**B**) Synergism evaluations for various drug combinations using Bliss combination index. Various drug combinations for different modules exhibited different values of synergism. (**C**) Synergism patterns of different modules with various drug combinations. White, synergy; Gray, additivity; Black, antagonism.

The relative efficacies of drug combinations for different modules were shown in Figure 6A. For module *i*, if the relative efficacy of drug combination is greater than 0, then the drug combination induced more reduction in integrated output compared to the basic module, vice versa. Different drug combinations and different modules exhibited different drug efficacy. For a specific module, some drug combinations had positive relative efficacy, indicating that this module allowed better performance of drug combinations than the basic module. Moreover, compared to the effects of single B-targeting drug (Figure 5), only a portion of drug targets in combination with B resulted in the increase of the relative drug efficacy (Table 1). This implies that, in order to improve the efficacy of the targeted therapy using drug combination, the appropriate drug targets should be chosen and combined.

**Table 1:** Comparing the relative efficacies of drug combinations to that of single B-targeting drug

	Drug targets combined with B			
	A	B	C	D
Module 1	▲	▼	▲	▲
Module 2	▲	▲	▲	▼
Module 3	▲	▲	▲	▼
Module 4	▼	▼	▼	▲
Module 5	▲	▲	▲	▼
Module 6	▼	▼	▼	▲
Module 7	▼	▼	▼	▲
Module 8	▼	▼	▼	▲

**Remark**: ▲ indicates that the drug combination increased the relative drug efficacy compared to the single B-targeting drug. Whereas ▼ indicates that the drug combination had lower relative drug efficacy than the single B-targeting drug.

We then quantitatively evaluated synergism for various drug combinations using Bliss combination index (see Materials and Methods). The values of combination index (CI) of different drug combinations used for modules 1–8 were computed (Figure 6B). The synergism patterns of different modules with various drug combinations were shown in Figure 6C, where white represented synergy (CI > 0.05), gray additivity (CI < − 0.05) and black antagonism (−0.05 < CI < 0.05). It should be noted that in this work 5% perturbation by noise is tolerated to separate the synergy and antagonism, taking into account of noise effects [37]. These results demonstrated that drug combinations in different modules exhibited differential synergism or antagonism, indicating that the topology of crosstalk played an important role in affecting the efficacy and synergism of drug combinations. In addition, we found that not all drug targets combined with B exhibited synergistic effects in various modules (Table 2).

**Table 2:** Synergisms of drug combinations with B-targeting drug

	Drug targets combined with B			
	A	B	C	D
Module 1	Additivity	Antagonism	Additivity	Synergy
Module 2	Additivity	Additivity	Additivity	Additivity
Module 3	Additivity	Antagonism	Antagonism	Synergy
Module 4	Additivity	Antagonism	Synergy	Synergy
Module 5	Antagonism	Antagonism	Antagonism	Additivity
Module 6	Synergy	Additivity	Additivity	Synergy
Module 7	Additivity	Antagonism	Synergy	Synergy
Module 8	Synergy	Antagonism	Additivity	Synergy

For modules 1, 4, 6, 7, and 8 that were resistant to the B-targeting drug, combining D with B as drug targets could not only increase the relative drug efficacies of these modules (Table 1) but also exhibit synergistic effects (Table 2). This indicates that the downstream proteins or transcriptional factors in two parallel pathways of crosstalk-linked signaling modules might be important potential targets of drug combination that effectively and synergistically reduces the resistance of targeted therapeutics.

### The impacts of strength of signaling crosstalk on the drug efficacy and synergism patterns

**Figure 7:**
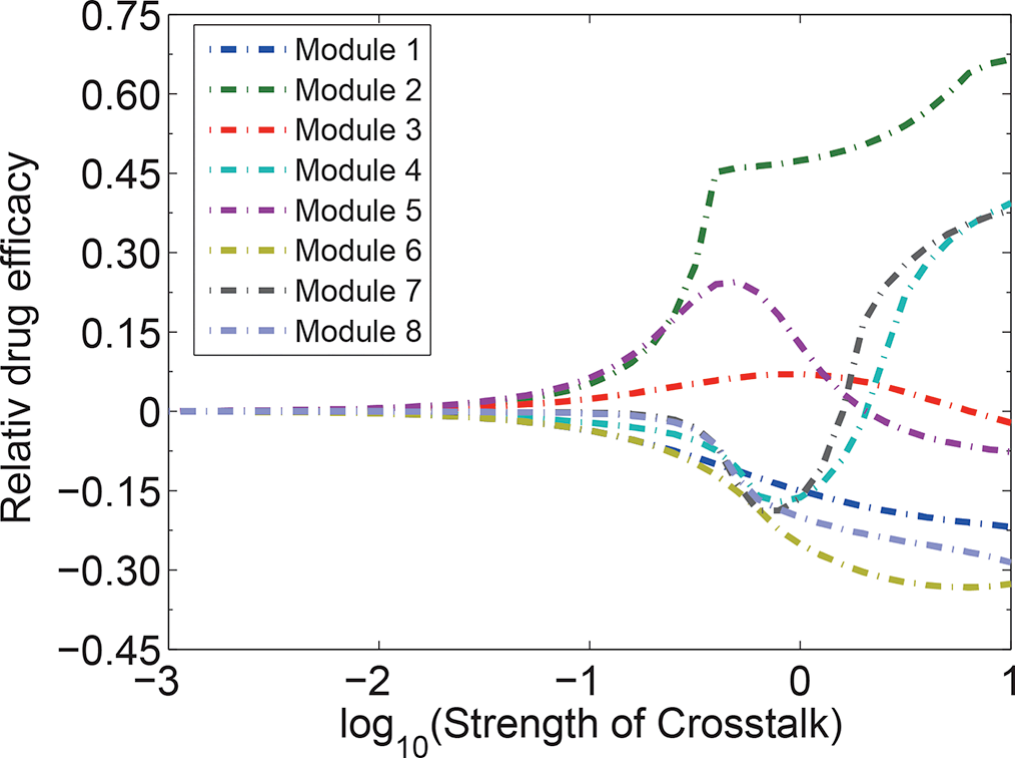
Impacts of the strength of signaling crosstalk on the relative drug efficacies of various modules The increasing strength of signaling crosstalk switches drug sensitivities of module 4, 5 and 7.

We then examined how the changes in the strength of signaling crosstalk in various modules affected the drug efficacy and synergism patterns of the drug combination. Our simulation demonstrated that the increasing strength of signaling crosstalk affected drug sensitivities, particularly for module 4, 5 and 7 (Figure 7). As the strength of crosstalk increased, modules 4 and 7 switched from drug-resistance to be drug-sensitive, whereas the mode of module 5 switched from drug-sensitive to drug-resistant. Furthermore, the increase in the strength of crosstalk enhanced the drug resistance in modules 1, 6 and 8 and amplified the drug sensitivity in module 2. The relative drug efficacy in module 3 was affected slightly by the strength of crosstalk.

**Figure 8:**
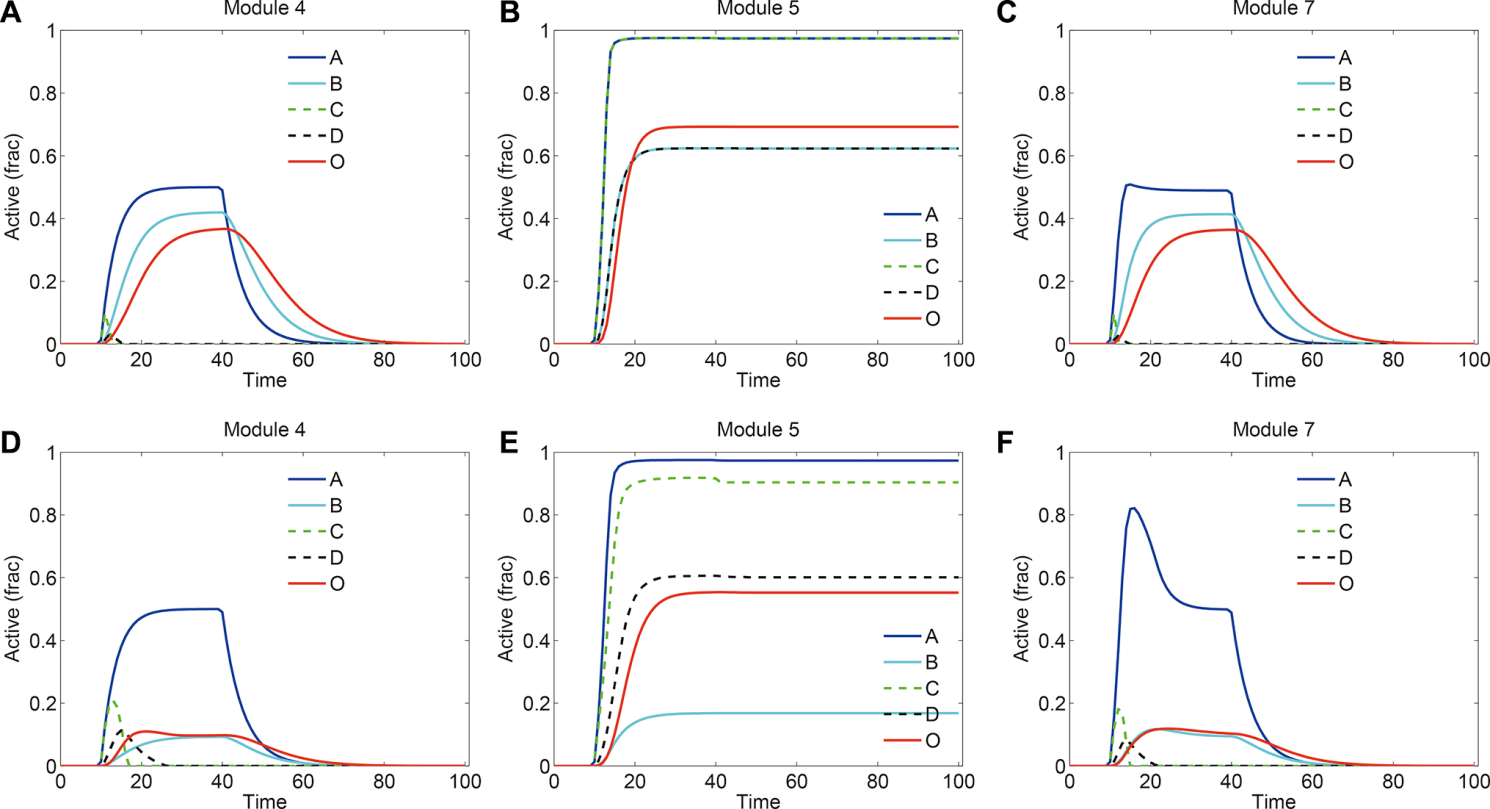
Signaling dynamics in modules 4, 5 and 7 with increased strengths of crosstalk The strength of crosstalk was set to 10-fold of the normal strength. Shown are signaling dynamics in modules 4, 5 and 7, respectively, (**A**–**C**) without and (**D**–**F**) with drug treatment. “Active (frac)” indicates fraction of activation of each protein.

We further investigated the signaling dynamics in modules 4, 5 and 7 with increased strengths of crosstalk (10 folds of the normal strength). The signaling kinetics in modules 4, 5 and 7 without and with drug treatment were shown in Figure 8A–8C and Figure 8D–8F, respectively. Compared to the normal situation in Figure 3, the stronger crosstalk inhibition of C by B in modules 4 and 7 resulted in more repression of C and D (Figure 8A, 8C). Following the drug treatment, in module 4 and 7 (Figure 8D), although B was decreased at a low level by drug treatment, C and D were still repressed by B due to the strong crosstalk inhibition. Therefore, in this situation, the signaling output was reduced more than that in normal situation (Figure 4) and, thus, more sensitive to the drug treatment. For module 5, the strong mutual activation between two pathways resulted in a more robust persistence of the signaling activation (Figure 8B). As such, the persistence of the signaling in module 5 was not lost when the strength of crosstalk was increased, which switched module 5 to be insensitive to the drug treatment.

## DISCUSSION

In this study, we analyzed the effects of signaling crosstalk on the efficacy and resistance of targeted drugs and drug combinations by employing module-based modeling approach. We demonstrated how the architecture of signaling crosstalk contributes to the drug resistance and analyzed the related signaling dynamics. We also identified the principles for selecting targets to effectively and synergistically reduce drug resistance using combinatorial drugs.

Recently, increasingly more attention has been paid to drug resistance to improve the efficacy of the targeted therapy for cancer patients. Several mechanisms have been proposed to account for the origin and acquisition of drug resistance. For example, at molecular scale, genetic and epigenetic modifications can render tumor cells insensitive to targeted drugs [23], resulting in drug resistance. At the cellular scale, tumor stem cells are thought to be left behind by chemotherapy although it kills most cells in a tumor, which might serve as an important mechanism of drug resistance [38, 39]. In addition, spatial intra-tumor heterogeneity might also contribute to the drug resistance [40, 41]. At the microenvironmental scale, due to the drug-induced secretion of various cytokines or growth factors, the dynamic microenvironment adaptation [42] also plays an important role in facilitating the ability of cancer cells to withstand therapeutic assaults [43]. Our study focused on the role of crosstalk among intracellular signaling pathways in drug resistance. We systematically verified the hypothesis that at molecular scale, in addition to genetic or epigenetic mutations, the dynamic adaptation in posttranslational activation of signaling pathways mediated by crosstalk might also render cancer cells to be insensitive to the drug treatment.

The topology of signaling network has been demonstrated to have profound impacts on the biological functions such as biochemical adaptation [44] and cell fate decision [45]. Jensen, K.J. et. al. [46] investigated the effect of the architecture of small molecular network on the drug efficacy and synergism. Charlebois, D. A et. al. [47] also demonstrated that coherent feedforward loop in transcriptional regulatory motifs could contribute to the drug resistance. Our work further revealed that some types of signaling crosstalk could also reduce the sensitivity of cancer cells to the drug inhibition. At the normal strength of crosstalk, a feedforward crosstalk in module 1 could yield drug resistance, which is consistent with the results in Ref. [47]. Moreover, our results also revealed some other crosstalk-linked modules (Modules 4, 6, 7, and 8) could also resist to the drug treatment. The predictions of modules 4 and 6 are supported by the experimental studies of drug resistance [19, 34].

Our model revealed a network architecture-dependent mechanism of signaling crosstalk-mediated drug resistance. The typical resistant modules (modules 1, 4, 6, 7, and 8) revealed by our model might be used to design robust modules in synthetic biology. Our model also predicted that the downstream proteins or transcriptional factors in two parallel pathways within crosstalk-linked signaling modules might be potential targets of drug combination that effectively and synergistically reduces the resistance of targeted therapeutics. The predicted synergistic effect of combination of B-targeting drug and D-targeting drug for module 4 is consistent with experimental studies [48, 49]. The principle revealed by our model might be useful for guiding the experimental design of drug combination.

With the increased strength of crosstalk, the relative efficacies and synergism patterns of drug combinations for different modules were shown in Figure S4. Compared to Figure 6, the increased strength of signaling crosstalk significantly influenced the synergism patterns of different modules. More specifically, the increase in strength of the crosstalk could trigger the switch of some signaling modules between drug sensitivity and drug resistance. For example, module 5, containing mutual crosstalk activation between two pathways, is drug-sensitive at lower strength of crosstalk but drug-resistant at higher strength of crosstalk. The mutual crosstalk activation could be viewed as positive feedback within the signaling network, which is able to maintain the persistence of the signaling activation of network as the strength of the crosstalk increases.

Feedback and feedforward loops in the signaling network have been demonstrated to significantly influence the drug effects [46]. In the future studies, we will extend our model to systematically investigate the effects of feedback and feedforward loops on the drug resistance and drug combinations. In addition, the drug administration of different dosages is very important but beyond the scope of this study that focuses on the effect of signaling crosstalk on drug response. In the future work, we will use the developed model to investigate the effect of drug dosages on the relative drug efficacies and synergism patterns of drug combinations for various signaling modules. Furthermore, we will try to study how to optimize dosing of drug combinations for different signaling modules.

As the signaling transduction and gene expression are often stochastic and noise exists extensively in the biochemical reaction networks [50], the effect of noise on the drug efficacy for various module structures should be examined to reveal how molecular noise interferes drug efficacy and contributes to drug resistance. In the future work, we are going to develop a stochastic model using chemical Langevian equations [51] for various signaling modules to simulate stochastic dynamics of molecules (e.g., proteins, transcriptional factors and genes). Such stochastic model might be helpful for our understanding on the causal relationship between the noise and the drug resistance.

In summary, our study investigated the role of signaling crosstalk in cell's adaptation to the drug treatment. Our study provides insight into the signaling crosstalk-mediated mechanisms underlying the drug resistance and delineates the implications associated with optimal targets of combination therapy for cancer patients.

## MATERIALS AND METHODS

### Computational modeling

According to the Michaelis-Menten kinetics [52], we formulated the following ordinary differential equations (ODEs) to model the kinetics of proteins in each module (Figure 2):

$$\frac{dX_{i}}{\text{dt}}=\sum_{j=1}^{5}\frac{V_{j,i}\cdot (1-X_{i})}{g_{i}\cdot K_{j,i}+(1-X_{i})}\cdot X_{j}-d_{i}\cdot X_{i}+f_{i}(t)$$ (1)

where *X_i_*, *i* = 1, 2, 3, 4, and 5, represents component A, B, C, D and output O in the signaling network, respectively. *V_j,i_* is the maximal activation rate of *X_i_* by *X_j_*, and *K_j,i_* is the corresponding Michealis constant. Different values of the matrix *V* characterize different module topologies. *d_i_* is the degradation rate of *X_i_*. *f_i_*(*t*) (*i* = 1 or 3) is an input function describing the effect of stimulus G1 on A or that of G2 on C, which is defined as follows,

$$f_{i}\left(t\right)=\{\begin{array}{ll} \frac{V_{A}\cdot G_{1}\left(t\right)}{K_{A}+G_{1}\left(t\right)}, & i=1;\\ \frac{V_{C}\cdot G_{2}\left(t\right)}{K_{C}+G_{2}\left(t\right)}, & i=3;\\ 0, & \text{Otherwise}. \end{array}$$ (2)

To incorporate the drug effects into the model, we defined a function $g_{i}=\left(1+\frac{\text{Drug}}{K_{\text{Di}}}\right)$ to multiply *K_j,i_* in Equation (1) if the drug inhibits *X_i_*, as in Ref. [30].

Concentrations and time in the model were non-dimensional and the total concentration normalized to 1. Standard parameter values were used to reproduce the key kinetic features of a realistic signaling network [16, 19] as shown in Figure 1. These values of parameters are listed below (for all *i* = 1, 2, …, 5 and *j* = 0, 1, …, 8):

*V_j, i_* = 0.4 if *X_j_* activates *X_i_*; *V_j, i_* = −1 if *X_j_* inhibits *X_i_*; *V_j, i_* = 0 if no link between *X_j_* and *X_i_*. *K_j,i_* = 0.8, *d_i_* = 0.2, *V_A_* = 0.2, *K_A_* = 0.5, *V_C_* = 0.2, *K_C_* = 0.5, *K_Di_* = 0.1.

The profiles of various types of stimuli (G1 and G2) are shown in Figure S2. In the typical simulation, a basic stimulus S0 was used for G1 and G2, while in the simulations with varied stimulus (Figure S3), S1–S6 for G1 and/or G2 were used. The above ODEs were numerically solved using 4-th order Runge-Kutta method. The *in silico* experiments was performed in MATLAB R2007b (MathWorks, USA).

### The relative drug efficacy

To evaluate the relative drug efficacy (*RDE*) for different modules, we defined the following index to quantify the relative decrease of integrated output (IO) due to the drug treatment in module *i* as compared to that in the basic module (module 0),

$$\text{RDE}\left(i\right)=\frac{IO_{\text{no}\_\text{drug}}^{\left(i\right)}-IO_{\text{drug}}^{\left(i\right)}}{IO_{\text{no}\_\text{drug}}^{\left(i\right)}}-\frac{IO_{\text{no}\_\text{drug}}^{\left(0\right)}-IO_{\text{drug}}^{\left(0\right)}}{IO_{\text{no}\_\text{drug}}^{\left(0\right)}}$$ (3)

where $IO_{\text{drug}}^{\left(i\right)}$ and $IO_{\text{no}\_\text{drug}}^{\left(i\right)}$ represent time-integrated output in the module *i* with or without drug treatment, respectively, that is, $IO^{\left(i\right)}=\int_{0}^{T}O^{\left(i\right)}\left(t\right)\text{dt}$, with T set as 100 in this work. If the relative drug efficacy is greater than 0 for module *i*, then the drug induced more reduction in integrated output of module *i* compared to the basic module, vice versa.

### Synergism evaluation of drug combination

Bliss combination index [53] was employed to evaluate the synergism between two drugs targeting “two” (same or different) components in the modules, which was defined as follows:

$$CI^{\left(i\right)}\left(d_{1},d_{2}\right)=R_{12}{}^{\left(i\right)}\left(d_{1},d_{2}\right)-\left(R_{1}{}^{\left(i\right)}\left(d_{1}\right)+R_{2}{}^{\left(i\right)}\left(d_{2}\right)-R_{1}{}^{\left(i\right)}\left(d_{1}\right)\cdot R_{2}{}^{\left(i\right)}\left(d_{2}\right)\right)$$ (4)

where $R_{12}^{\left(i\right)}\left(d_{1},d_{2}\right)$ is relative ratio of integrated output reduced by the combinatorial drug 1 and drug 2 to that without drug treatment in the module *i*. $R_{12}^{\left(i\right)}\left(d_{1},d_{2}\right)$ was computed as

$$R_{12}{}^{\left(i\right)}\left(d_{1},d_{2}\right)=\frac{IO^{\left(i\right)}\left(d_{1},d_{2}\right)-IO_{\text{no}\_\text{drug}}^{\left(i\right)}}{IO_{\text{no}\_\text{drug}}^{\left(i\right)}}.$$ (5)

Similarly, $R_{1}^{\left(i\right)}\left(d_{1}\right)$ or $R_{1}^{\left(i\right)}\left(d_{2}\right)$ is relative ratio of integrated output under the treatment of single drug 1 or drug 2 compared to that without drug treatment in the module *i*. $R_{1}^{\left(i\right)}\left(d_{1}\right)+R_{2}^{\left(i\right)}\left(d_{2}\right)-R_{1}^{\left(i\right)}\left(d_{1}\right)\cdot R_{2}^{\left(i\right)}\left(d_{2}\right)$ in Equation (4) models the expected effect of two combinatorial drugs [53].

If the combination index is greater than 0, the combination effect of the drug 1 and drug 2 was evaluated to be synergistic, while if this index is less than 0, these two drugs were considered to be antagonistic, otherwise additive.

## SUPPLEMENTARY MATERIALS FIGURES


