# Supplementary material for: Modeling of signaling crosstalk-mediated drug resistance and its implications on drug combination
Source: Oncotarget. 2016 Aug 31;7(39):63995–4006. doi: 10.18632/oncotarget.11745 (PMC5325420; doi:10.18632/oncotarget.11745)
Supplement: Supplementary file 1 [file oncotarget-07-63995-s001.pdf]

Modeling of signaling crosstalk-mediated drug resistance and its implications on drug combination

Supplementary Materials

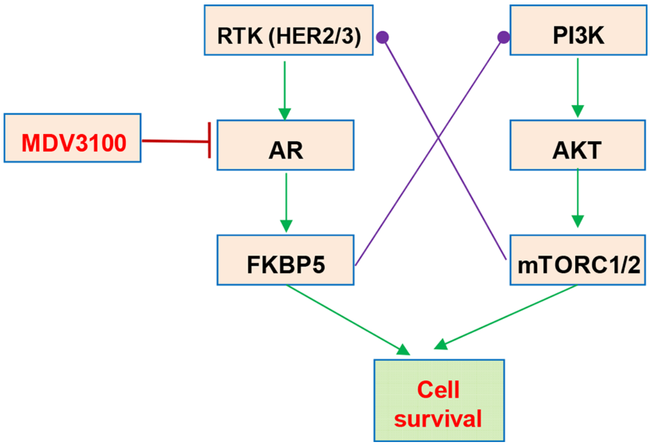

Supplementary Figure S1: A realistic case of signaling module with mutual crosstalk inhibition that renders prostate cancer cells to be resistant to the AR inhibitor MDV3100 [34], which is consistent with our prediction of module 6.

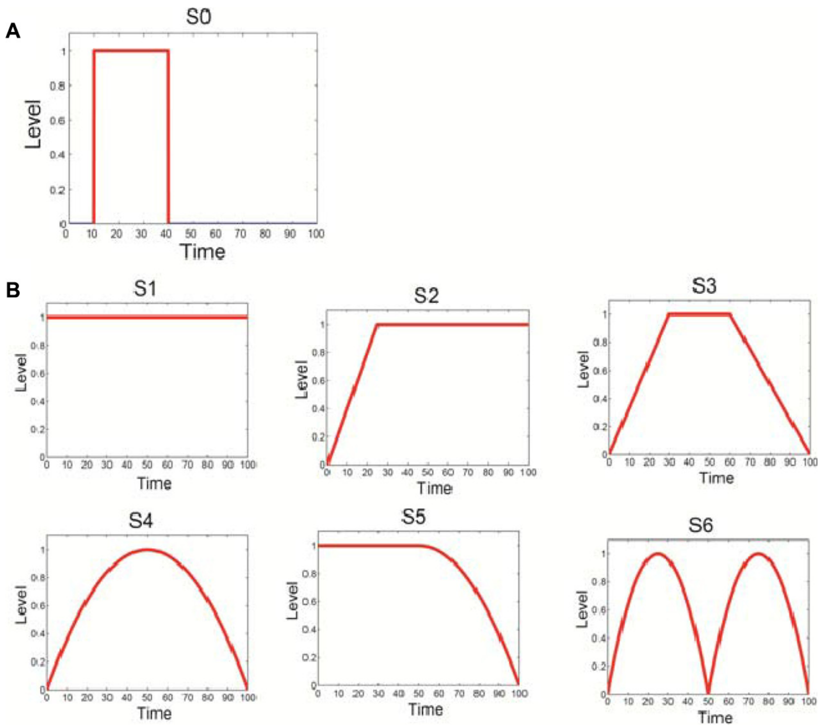

Supplementary Figure S2: Profiles of various stimuli used in the simulation. (**A**) Basic stimulus. (**B**) Variant types of stimulus.

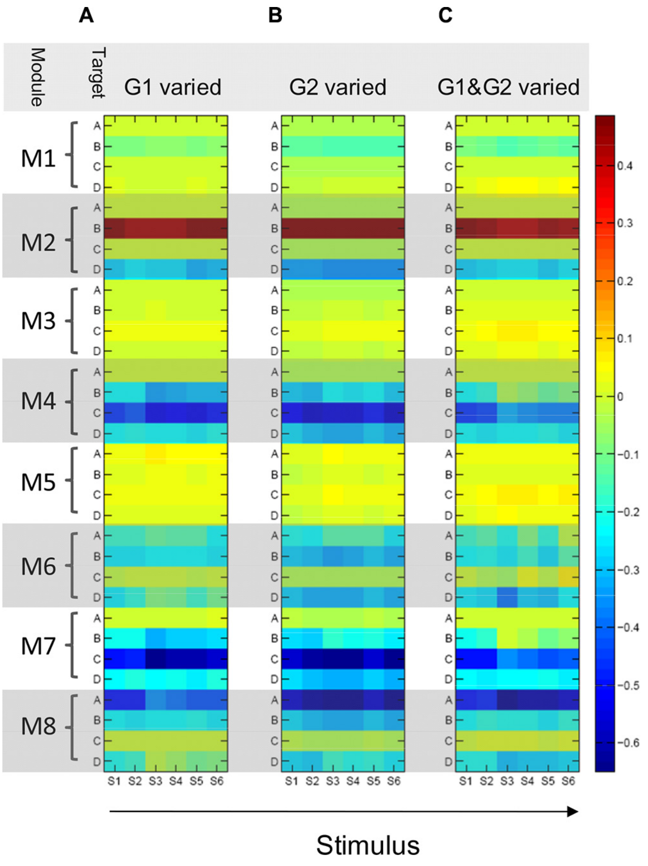

Supplementary Figure S3: Efficacies of drugs targeting different components in the modules 1-8 in response to various stimuli (S1 – S6). (**A**) Input G1 was varied to S1, S2 … or S6, and G2 was S0. (**B**) Input G2 was varied to S1, S2 … or S6, and G1 was S0. (**C**) Both input G1 and G2 were varied. Different modules (M1, M2, …, and M8) and different targets (A, B, C and D) exhibited different relative drug efficacies compared to the basic module (M0).

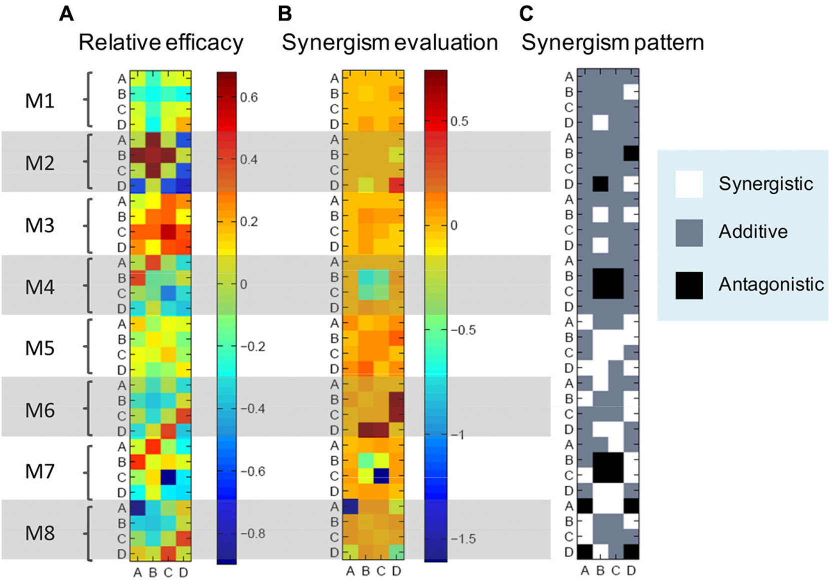

Supplementary Figure S4: Drug combination evaluation for different modules with increased strengths of signaling crosstalk. (**A**) Relative efficacies of drug combinations targeting two (different or same) components (A, B, C and D) for different modules. (**B**) Synergism evaluations for various drug combinations using Bliss combination index. (**C**) Synergism patterns of different modules with various drug combinations. Compared to Figure 6, the increased strength of signaling crosstalk significantly influenced the synergism patterns of signaling modules.
